# Risk factors associated with infection recurrence of posttraumatic osteomyelitis treated with Ilizarov bone transport technique—a retrospective study of 149 cases

**DOI:** 10.1186/s12891-021-04430-2

**Published:** 2021-06-23

**Authors:** Ainizier Yalikun, Maimaiaili Yushan, Wenqiang Li, Alimujiang Abulaiti, Aihemaitijiang Yusufu

**Affiliations:** 1 Department of Microrepair and Reconstruction, The First Affiliated Hospital of Xinjiang Medical University, Urumqi, Xinjiang, China; 2 No.2 Department of orthopedics surgery, The Friendship Hospital of Yili Kazakh Autonomous Prefecture, Xinjiang, China

**Keywords:** Posttraumatic osteomyelitis, Recurrence, Risk factors;bone transport

## Abstract

**Background:**

Post-traumatic tibial osteomyelitis is considered as complex clinical problem due to its unique characteristics such as prolonged course, multi-staged treatment and high recurrence rate. The purpose of this study is to identify and analyze the causes and risk factors associated with infection recurrence of tibial osteomyelitis treated with Ilizarov technique.

**Methods:**

From January 2011 to January 2019, a total of 149 patients with post-traumatic tibial osteomyelitis treated with Ilizarov bone transport technique were included in this study. Demographic and clinical data were collected and analyzed. Univariate analysis and logistic regression analysis were used to analyze the factors that may affect the recurrence or reinfection of post-traumatic tibial osteomyelitis after treated with Ilizarov bone transport technique.

**Results:**

All included patients were successfully followed up with an average of 37.5 month (18–78 month), among them, 17 patients (11.4%) occurred with recurrence or reinfection of tibial osteomyelitis in which 2 cases were in distraction area and 15 cases in docking site. Among them, 5 patients were treated successfully with appropriate intravenous antibiotic, the remaining 12 patients were intervened by surgical debridement or bone grafting after debridement. Univariate analysis showed that *Pseudomonas aeruginosa* infection, bone exposure, number of previous operations (> 3 times), blood transfusion during bone transport surgery, course of osteomyelitis > 3 months, diabetes was associated with recurrence or reinfection of postoperative tibial osteomyelitis. According to the results of logistic regression analysis, *Pseudomonas aeruginosa* infection, bone exposure, and the number of previous operations (> 3 times) are risk factors for recurrence or reinfection of posttraumatic tibial osteomyelitis treated with Ilizarov bone transport technique, with odds ratios (OR) of 6.055, 7.413, and 1.753, respectively.

**Conclusion:**

The number of previous operations (> 3 times), bone exposure, and *Pseudomonas aeruginosa* infection are risk factors for infection recurrence of posttraumatic tibial osteomyelitis treated with Ilizarov bone transport technique.

## Background

Post-traumatic tibial osteomyelitis is considered as complex clinical problem due to its unique characteristics such as prolonged clinical course, multi-staged treatment, and high recurrence rate [1]. High energy accompanied with open injury leads to severe bone and soft tissue contamination which requires thorough debridement [2], on the contrary, repeated debridement will cause more serious bone loss and defects which result in further complicated situations such as chronic infections, soft tissue defects, joint contractures, and lower limb deformities or discrepancy [3–5]. At present, Ilizarov bone transport technique has become the preferred treatment option to simultaneously solve this series of problems [6]. Ilizarov first introduced the successful treatment of bone defects through distraction osteogenesis technique in 1969, that is, bone segment transported through external fixation devices [7, 8]. Since the 1990s, this technique has become a globally accepted treatment option for the treatment of bone defects caused by infection [9]. The concept of bone regeneration using Ilizarov technique is the cornerstone of the contemporary bone defect reconstruction surgery [10] .

Contrary to the data showing the increasing incidence of posttraumatic osteomyelitis, there are few studies focusing on the risk factors of recurrence or reinfection of post-traumatic osteomyelitis treated with Ilizarov bone transport technique [11]; The purpose of this study is to identify and analyze the causes and risk factors associated with infection recurrence of tibial posttraumatic osteomyelitis treated with Ilizarov technique.

## Methods

### Patients

This retrospective study was approved by the Ethics Committee of our institution and a total of 149 cases with post-traumatic tibial osteomyelitis treated with the Ilizarov bone transport technique from January 2011 to January 2019 were included. Inclusion criteria: [1]. Age 18 to 65 years [2];. Patients diagnosed with posttraumatic tibial osteomyelitis and treated with Ilizarov single or double-level bone transport technique using circular or mono-lateral external fixator [3]. bone defects ≥3 cm and minimal follow up is more than 18 month after removal of external fixator with good compliance. Exclusion criteria: [1]. Bone defect < 3 cm or bone defect accompanied by adjacent joint infection [2];. Complicated with severe cardiovascular and cerebrovascular disease, mental disease, liver and kidney dysfunction [3];. Autoimmune disease, blood disease and serious Osteoporosis [4];. Poor compliance and loss of follow-up.

### Definition of posttraumatic osteomyelitis(PTO)

PTO was defined according to the guidelines and standards of the Centers for Disease Control and Prevention (CDC)/National Health Care Safety Net (NHSN), requiring at least one of the following standards: [1] Any microorganisms are cultured from bone and soft tissue [2];. General appearance of tissue or histopathological examination shows evidence of osteomyelitis; and contains at least 2 of the following signs and symptoms of inflammation: fever(> 38.0 °C), swelling, pain, redness, fever, exudation, delayed wounds healing with bone or plate exposure; plus at least one of the following: a) positive blood culture or no blood culture but imaging results suggest microbial infection, if not clear, it will be confirmed by clinically relevant symptoms; b) imaging evidence suggests infection, If it is not clear, it is confirmed by clinically relevant symptoms [12].

### Treatment

## Preoperative preparation

X-ray, computed tomography scans and MRI of the affect limb was examined before operation to evaluate the extent of infection or dead bone. Laboratory tests include CRP (C-reactive protein), ESR (erythrocyte sedimentation rate) and WBC (White blood cell count). The type of external fixator device (circular or monolateral) was decided based on the imaging result and surrounding soft tissue condition of the affected limb.

## Surgical procedure

A complete removal of hardware, radical debridement of all necrotic and infected bone and soft tissue, and/or implantation of an antibiotic-impregnated cement spacer to improve stability were performed prior to bone transport. Cortical bleeding, described as the so-called “paprika sign”, was accepted as an indication of vital osseous and ensure that the medullary cavity is recanalized. Tissue specimens from six different area were taken and sent for bacterial culture and drug susceptibility tests to guide the surgeon for the appropriate postoperative antibiotics. The wound was irrigated by using hydrogen peroxide, mucosal iodine solution, and physiological saline repeatedly, use local tissue flaps or tension-free sutures to repair small soft tissue defects, use flap transfer or free skin grafts to cover soft tissue defects.

External fixator was mounted according to the length of the bone defect and the surrounding soft tissue condition, minimal invasive Gigli saw osteotomy was applied to protect the periosteum as much as possible. For bone defects larger than 8 cm or more than 40% of the original bone length, bifocal (double level) bone transport was adopted.

## Postoperative management and follow-up

Intravenous appropriate antibiotics was administered for at least 6 weeks or until ESR and CRP levels return to normal based on the result of bacterial culture and drug susceptibility test. Passive knee and ankle exercises are started on the second day after surgery to encourage early partial weight-bearing. Bone transport was initiated 7–10 days after surgery. For patients with flap transfer, bone transport was started after the healing of the flaps which usually takes 2–3 weeks. For single level bone transport, fragment was transported at a rate of 0.25 mm four times per day. For double level bone transport, if bone transport in the same direction (proximal to distal), the fragment near the bone defect was transported at a rate of 0.5 mm four times per day, and another fragment far from the defect was transported 0.25 mm four times per day. If bone transport in the opposite direction, each fragment on both sides of the bone defect proceeded at a rate of 0.25 mm four times per day.

The patients were followed up in the outpatient clinic every 2 weeks, and physical and radiographic examinations were performed to assess pin track condition, external fixator stability and adjacent joint range of motion. When the bridging callus appeared radiographically and limb length equalization was achieved, the frame was dynamized in order to assess the mechanical stability of the regenerated bone and then removed as a daycare procedure. At the time of removal of the external fixator, the leg was protected in a long-leg cast or cast-brace for 4 to 6 weeks with the patient using only partial weight-bearing.

### Definition of recurrence of osteomyelitis

Recurrence refers to the recurrence of symptoms and signs of osteomyelitis, plus one or more positive bone cultures isolated from previous treatments [13].

### Data collection

Collection of demographic and clinical data include gender, age, smoking, diabetes, initial injury (open or closed), index surgical fixation method (internal or external fixation), osteomyelitis course (> 3 months or ≤ 3 months), the number of previous operations, the presence or absence of bone exposure, preoperative laboratory examinations (CRP, ESR, WBC), the type of external fixation (circular or monolateral), the size of the bone defect, the type of bone transport (single or double level), whether there is flap coverage or blood transfusion during the operation, microbiological examination, time of recurrence or reinfection.

### Statistical analysis

Continuous variables (age, bone defect length, number of previous operations, etc.) were compared by using t-tests, and Pearson’s chi-square test or Fisher-exact test was used to compare categorical variables (gender, smoking, and diabetes). The variables with statistical differences in *p* < 0.05 in univariate analysis were brought into the binary logistic regression analysis for analysis of related risk factors; the results *p* < 0.05 had differences. SPSS version 22.0 (IBM Corp, USA) was used to analyze all data.

## Result

The study include 149 patients with post-traumatic tibial osteomyelitis, 118 (79.2%) men and 31 (20.8%) women, with an average age of 39.3 (19–61) years and an average follow-up was 37.6 (18–78) months; the injury mechanism include 129 cases (86.6%) of open fractures, 20 cases (13.4%) of close fractures; the index surgery include 67 cases (45.0%) with external fixation, and 82 cases (55.0%) with internal fixation. The average bone defect size was 6.9 ± 1.9 cm. The bone transport was conducted in 102 cases (68.5%) by using ring external fixator and 47 cases (31.5%) by using monolateral external fixator. 109 cases (73.2%) with single-level bone transport and 40 cases (26.8%) with double-level bone transport. There were 47 cases (31.5%) with smoking history, 23 cases (15.4%) had diabetes, and 62 cases (41.6%) had bone exposure.

**Fig. 1:**
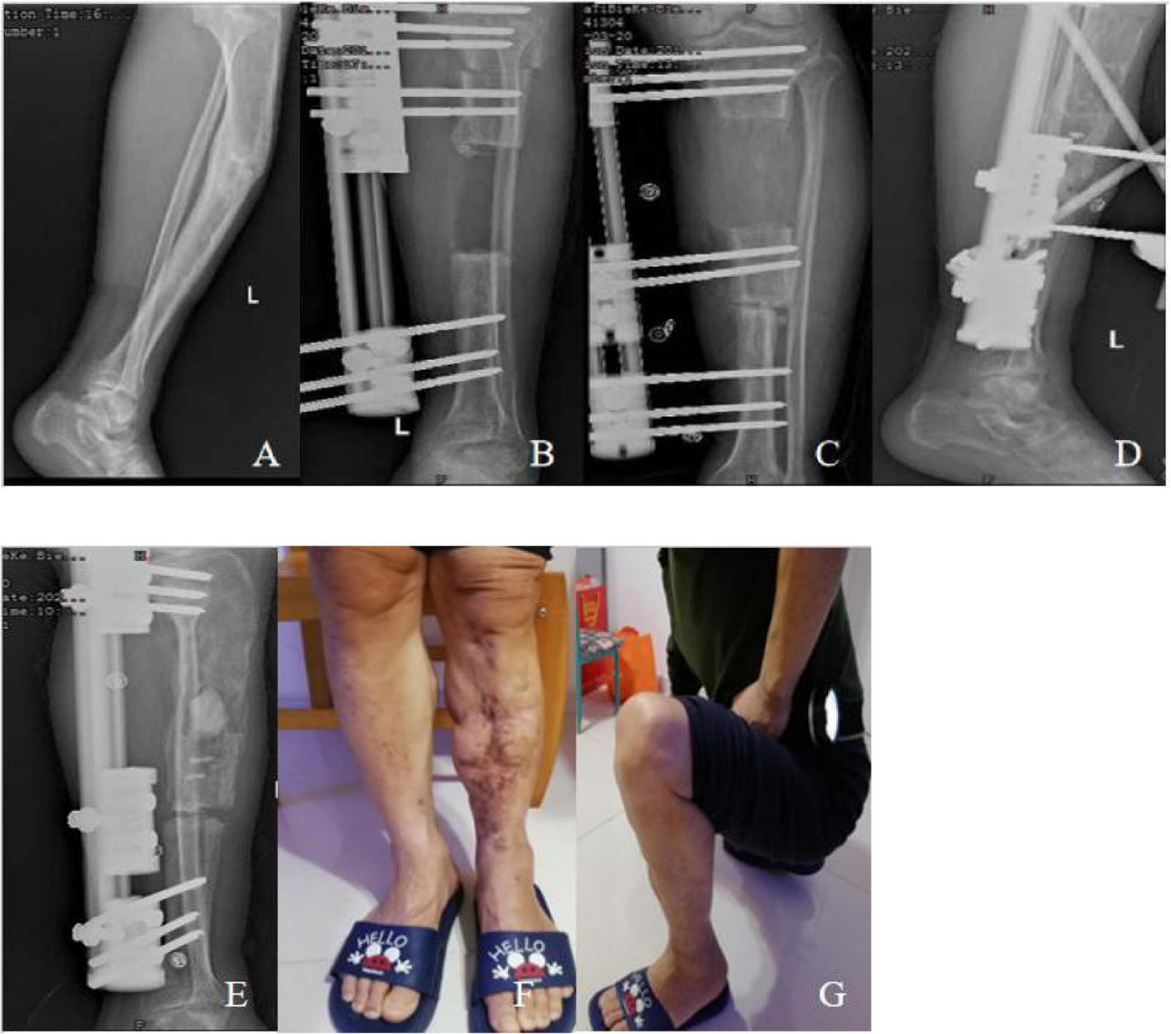
A male patient with left tibia fracture caused by traffic accident and post-traumatic osteomyelitis after plate internal fixation.(**A**). X-ray film at 12 months after removal of the internal fixator showing tibia osteomyelitis; (**B**). Anteroposterior X-ray films on the day after radical debridement and installation of external fixation; (**C**) Anteroposterior X-ray films at 119 days after operation, showing bone contact was reached; (**D**). Lateral X-ray films at 11.5 months after operation shows good regenerate consolidation,and bone grafting at the docking site.(**E**). After 9 months removed external fixation, infection recurrence were located in the distraction region .The patient suffered refracture at the docking site at the same time, which were treated with debridement, antibiotic cement and extenal fixation.(**F,G**) Function recovery at 19 months after removal of external fixator

Among 17 patients (11.4%) with infection recurrence which include 14 males and 3 females. The average time of recurrent infection after removal of the external fixation was 10.8 ± 3.0 (3.1–17.3) months, 5 of them (29.4%) received intravenous antibiotic treatment and the remaining 12 cases (70.6%) underwent debridement with or without bone grafting, and all recurrent infection were cured successfully. In 2 cases (13.3%), infection recurrence were located in the distraction region which was successfully treated after intravenous antibiotic treatment, infection recurrence was located at the docking site in 15 cases (86.7%), of which 3 cases were treated with intravenous antibiotics, and 12 cases were successfully treated with debridement with or without bone grafting.(Typical case see Fig.1).

In this study, the positive rate of microbial culture in all patients was 81.9% (122/149), of which 63 cases (51.7%) of *Staphylococcus aureus* (including methicillin-resistant *Staphylococcus aureus* MRSA) and *Pseudomonas aeruginosa* 32 Cases (26.2%), 15 cases of *Escherichia coli* (12.3%), 7 cases of Acinetobacter baumannii (5.7%), 5 cases of *Klebsiella pneumoniae* (4.1%); the positive rate of microbial culture in patients with recurrent infection was 88.2% (15/17), 5 cases of *Staphylococcus aureus* (including MRSA) (33.3%), 9 cases of *Pseudomonas aeruginosa* (60.0%), and 1 case of *E. coli* (6.7%).

### Factors related to recurrent infection

Among the univariate variables, there was a statistical difference between the infection recurrence group and the non-recurrence group in clinical course of osteomyelitis> 3 months (94.1%vs67.4%, *p* = 0.023), intraoperative blood transfusion (52.9%vs22.7%, *p* = 0.018), bone exposure (88.2%vs35.6%, *p* < 0.001), diabetes (35.3%vs12.9%, *p* = 0.04), the number of previous operations (3.9 ± 1.3vs2.6 ± 1.4, *p* < 0.001), *Pseudomonas aeruginosa* infection (52.9% vs 17.4%, *p* = 0.002). On the contrary, there was no statistical difference between the two groups in the remaining variables. (See Table 1).

**Table 1:** Stating the recurrence/non-recurrence refers to infection

Demographic data	Recurrence (*n* = 17)	Non-recurrence(*n* = 132)	*p*-value
**Sex**			
Male/Female	14(82.4%)/3(17.6%)	104(78.8%)/28(21.2%)	0.981
**Age**	41.9 ± 9.2	39.0 ± 6.1	0.081
**Smoking**	5(29.4%)	42(31.8%)	0.841
**Diabetes**	6(35.3%)	17(12.9%)	0.040
**Initial injury**			
Open/ Close	15(88.2%)/2(11.8%)	114(86.4%)/18(13.6%)	1.000
**Index surgery fixation**			
Internal /External fixation	10(58.8%)/7(41.2%)	72(54.5%)/60(45.5%)	0.739
**Course of osteomyelitis**			
(> 3 months)	16(94.1%)	89(67.4%)	0.023
**Previous Number of Surgeries**	3.9 ± 1.3	2.6 ± 1.4	< 0.001
**Bone exposure**	15(88.2%)	47(35.6%)	< 0.001
**Preoperative Lab result**			
"WBC” and “ESR/CRP”	7.6 ± 2.1and25.1 ± 3.7/11.3 ± 2.7	8.1 ± 2.4and23.6 ± 3.4/10.9 ± 2.2	0.496and0.082/0.524
**Type of external fixator**			
Ring/Monolateral	13(76.5%)/4(23.5%)	89(67.4%)/43(32.6%)	0.450
Size of bone defect	6.6 ± 1.9	7.0 ± 2.0	0.441
**Level of bone transport**			
Single/Double	13(76.5%)/4(23.5%)	92(69.7%)/40(30.3%)	0.564
**Flap coverage**	4(23.5%)	25(18.9%)	0.901
**Intraoperative blood transfusion**	9(52.9%)	30(22.7%)	0.018
**Microbiological result**			
*Pseudomonas aeruginosa*	9 (52.9%)	23 (17.4%)	0.002
*Staphylococcus aureus* (include MRSA)	5(29.4%)	58(43.9%)	0.254

**WBC**:white blood count; **CRP**:C-reactive protein; **ESR**:erythrocyte sedimentation rate; **MRSA**: methicillin-resistant Staphylococcus aureus

Among the 17 patients with recurrent infection, 12 cases were treated with surgery and 5 cases were treated with antibiotics only. There was a statistical difference between the operation and non-operation group in the time of infection reoccurrence (9.9 ± 2.8vs13.0 ± 2.4, *p* = 0.043) and *Pseudomonas aeruginosa* infection (75%vs0%, *p* = 0.022), whether it is infected with *Pseudomonas aeruginosa* is statistically different in the recurrence time (9.1 ± 2.9vs12.7 ± 1.9, *p* = 0.009). (See Table 2).

**Table 2:** Comparison of operative and non-operative group

Demographic data	Surgical treatment(*n* = 12)	No surgical treatment(*n* = 5)	p-value
**Sex**			
Male/Female	10(83.3%)/2(16.7%)	4(80.0%)/1(20.0%)	1.000
**Age**	42.4 ± 9.7	40.6 ± 8.6	0.722
**Smoking**	4(33.3%)	1(20.0%)	1.000
**Diabetes**	5(41.7%)	1(20.0%)	0.600
**Course of osteomyelitis**			
(> 3 months)	11(91.7%)	5(100.0%)	1.000
**Previous operation time**	4.0 ± 1.5	3.8 ± 0.8	0.782
**Bone exposure**	11(91.7%)	4(80.0%)	0.515
**Preoperative Lab result**			
WBC and ESR/CRP	7.5 ± 2.4and 4.3 ± 4.2/11.1 ± 3.1	8.0 ± 1.5and 25.5 ± 1.3/11.8 ± 1.8	0.657and 0.555/0.657
**Type of external fixator**			
Ring/Monolateral	9(75.0%)/3(25.0%)	4 (80.0%)/1(20.0%)	0.538
**Size of bone defect**	6.2 ± 1.6	7.3 ± 2.3	0.268
**Average recurrent time (months)**	9.9 ± 2.8	13.0 ± 2.4	0.043
**Level of bone transport**			
Single/Double	10(83.3%)/2(16.7%)	3(60.0%)/2(40.0%)	1.000
**Flap coverage**	1(8.3%)	3(60.0%)	0.053
**Intraoperative blood transfusion**	6(50.0%)	3(60.0%)	1.000
**Microbiological result**			
Pseudomonas aeruginosa	9 (75.0%)	0 (0%)	0.009
Staphylococcus aureus (include MRSA)	3(25.0%)	2(40.0%)	0.600

**WBC**:white blood count; **CRP**:C-reactive protein; **ESR**:erythrocyte sedimentation rate; **MRSA**: methicillin-resistant Staphylococcus aureus

### Analysis of risk factors related to infection recurrence

Logistic regression analysis showed that the course of osteomyelitis> 3 months, intraoperative blood transfusion, and diabetes were not risk factors for infection recurrence. The number of previous operations (repeated operations), bone exposure, and *Pseudomonas aeruginosa* infection were risk factors for recurrent infection, and the OR values were 1.753, 7.413 and 6.055, respectively. (See Table 3).

**Table 3:** Risk factors of posttraumatic osteomyelitis

Variables	OR value	***P*** value
Diabetes	4.225	0.080
clinical course of osteomyelitis> 3 months	4.753	0.173
Intraoperative blood transfusion	2.880	0.130
Bone exposure	7.413	0.020
Previous operation time	1.753	0.018
Pseudomonas aeruginosa infection	6.055	0.012

## Discussion

The treatment of recurrent infection after posttraumatic tibial osteomyelitis may need repeated debridement, prolong treatment time and even bring a huge burden to patients and society [14, 15]. However, research on the risk factors of recurrent infection after surgery is rare. Studies have showed that the infection recurrence rate after treatment of posttraumatic tibial osteomyelitis with bone defect can reached to 10–20% [16]. In this study, infection recurrence rate in the treatment of post-traumatic tibial osteomyelitis by using Ilizarov technique was 11.4%, which was consisted with previously reported study [17], which also confirms that the Ilizarov technique is safe and effective treatment option in treatment of post-traumatic tibial osteomyelitis. This study shows that the number of previous operations (repeated operations), bone exposure, and *Pseudomonas aeruginosa* infection are risk factors for recurrent infection.

The important principle of eradication of osteomyelitis is to thoroughly and adequately debride the infected bone and soft tissues until there is a bit of bleeding active bone (paprika sign) [18]; the severity of the trauma, the patient’s immune status, and incomplete debridement are all possible to put patient at a high risk of additional surgery. According to our study, the number of previous operations (repeated operations) is a risk factor for infection reoccurrence of post-traumatic osteomyelitis, which is consisted to other studies results [19, 20]. First of all, repeated operations will prolong the hospital stay, additional damage to bone and soft tissue, increase pain to impaired limb function, which will affect the quality of life of patients [21]. In addition, repeated operations may produce more scar tissue and affect the flexibility and blood supply of the soft tissues, which makes subsequent surgical exposure difficult, and even causes adverse outcomes such as recurrent infection [22]. Therefore, carefully designing surgical treatment plans, performing limb reconstruction by experienced professional teams, and minimizing additional surgical operations have become particularly important in the treatment of post-traumatic osteomyelitis.

Studies have shown that the recurrence rate of osteomyelitis caused by *Pseudomonas aeruginosa* infection is 2 times higher compared with *Staphylococcus aureus* [23]. Compared with any other isolated pathogens, the prognosis is poor, and it is positively correlated with amputation. Among the 17 cases of infection recurrence in this study, 9 cases of *Pseudomonas aeruginosa* (52.9%), 5 cases (29.4%) of *Staphylococcus aureus* (including MRSA); *Pseudomonas aeruginosa* infection rate was higher than other cases. Bacterial infections are mainly caused by the ability of *Pseudomonas aeruginosa* to form biofilms. It is also recognized as an important cause of chronic infections. Bacteria exist in aggregates wrapped in the extracellular matrix produced by themselves, which shows resistance to antibacterial drugs. Tolerance and drug resistance are difficult to eradicate with antibiotic treatment [24]. Secondly, *Pseudomonas aeruginosa* has a protective effect in infection, and it has been proven that it can eliminate free radicals released by activated macrophages in vitro and prevent itself from being phagocytosed and cleared [25]. Recent studies have suggested that *Pseudomonas aeruginosa* is directly related to the recurrence of post-traumatic osteomyelitis infection [26]. In summary, in charge surgeon need to be alerted to patients with posttraumatic tibial osteomyelitis caused by *Pseudomonas aeruginosa* infection.

In this study, the bone exposure in cases with infection recurrence was as high as 88.2% (15/17), which strongly indicated that the complete coverage of soft tissue is of great significance for infection control and prevention. Early coverage of soft tissue can improve local blood supply, provide nutrition, eliminate dead space, promote local immune defense and the effectiveness of antibiotic administration [27]. Therefore, while dealing with severe open fractures and thoroughly debridement to eradicate infection, the reasonable reconstruction of the surrounding soft tissue is still a very concern.

Our study showed that the course of osteomyelitis> 3 months, intraoperative blood transfusion, and diabetes are related factors for the recurrence of post-traumatic osteomyelitis infection. If post-traumatic osteomyelitis is not treated effectively in time, it may cause more serious infections over time, and even cause larger dead bones and deep soft tissue infection, laying hidden source for recurrent infection. A study analyzed the risk factors of 116 cases of recurrence of osteomyelitis infection, which concluded that the course of osteomyelitis> 3 months is directly related to the recurrence of osteomyelitis [28]. In this study, 52.9% (9/17) of patients with recurrent infection received intraoperative blood transfusion, which was much higher than 22.7% (30/132) of the non-infection recurrence group. In an analysis of 192 patients with post-traumatic osteomyelitis, risk factors for recurrent infection were analyzed which showed that the risk of infection recurrence in patients receiving blood transfusion was 2 times higher [26], which is similar to our study. Another study proposed that the risk of multi-microbial infection in patients undergoing blood transfusion during orthopedic surgery is 2.15 times higher than that of patients without blood transfusion [29]. Intraoperative blood transfusion may reflect the severity of the injury, the complexity of the operation and even the longer operation time. Therefore, more attention was given to patients with intraoperative blood transfusion. In this study, patients with diabetes accounted for 15.4% (23/149), 35.3% (6/17) in the infection recurrence group, and 12.9% (17/132) in the non- infection recurrence group. Tice and Lin et al. [30, 31] have confirm that diabetes is a related factor for the recurrence of osteomyelitis. Therefore, we recommend that blood glucose should be strictly controlled during the perioperative period and before the external fixator is removed after the operation in patients with posttraumatic tibial osteomyelitis accompanied with diabetes, and multidisciplinary cooperation with consultant to endocrinologists is strongly recommended.

Among 17 cases with recurrent infection, recurrence time of infection was shorter in non-operative group than operative group (*p* = 0.043), and all recurrent infection cases with *Pseudomonas aeruginosa* were underwent surgical intervention. Age, smoking, bone defect size, intraoperative flap coverage, bone transport level (single and double level), external fixator type (ring or monolateral), initial fixation type (internal fixation, external fixation), initial injury mechanism (open or close), laboratory tests (erythrocyte sedimentation rate, white blood cells, C-reactive protein) and other factors have no significant influence on the recurrent infection.

## Conclusion

The number of previous operations (repeated operations), bone exposure, and *Pseudomonas aeruginosa* infection are risk factors for infection recurrence of posttraumatic tibial osteomyelitis treated with Ilizarov technique. Blood transfusion during bone transport procedure, osteomyelitis course> 3 months, diabetes may be related to post-traumatic tibial osteomyelitis infection recurrence. Ilizarov technique is an effective method in the treatment of posttraumatic tibial osteomyelitis.

## Data Availability

The datasets analysed during the current study are available from the corresponding author on reasonable request. This retrospective study was approved by the Ethics Committee of our institution.

## Abbreviations

PTO: posttraumatic osteomyelitis
CRP: C-reactive protein
ESR: erythrocyte sedimentation rate
WBC: White blood cell count
